# Dual role of complement in neuronal repair

**DOI:** 10.3389/fimmu.2025.1712452

**Published:** 2025-12-03

**Authors:** Agnieszka Lukomska, Peter Ciesielski, Mariusz Z. Ratajczak, Magdalena Kucia

**Affiliations:** 1 Department of Regenerative Medicine, Center for Preclinical Studies and Technology, Medical University of Warsaw, Warsaw, Poland; 2 Stem Cell Institute at Graham Brown Cancer Center, University of Louisville, Louisville, KY, United States

**Keywords:** complosome, axon regeneration, C3aR, C5aR, NLRP3, mTOR

## Abstract

The complement system, long regarded as an arm of innate immunity, is now recognized as an important modulator of nervous system pathophysiology. Following acute injury or in chronic neurodegenerative diseases, promoting neuronal survival and axon regeneration remains a formidable clinical challenge. This review synthesizes the extensive, paradoxical evidence of complement’s dual role in neurodegeneration and repair. We examine how complement activation is both detrimental—driving neuroinflammation, apoptosis, and pathological autophagy via receptors like C5aR1 and its interaction with the NLRP3 inflammasome—and beneficial, promoting C5a-mediated phagocyte recruitment for debris clearance and C3-dependent synaptic stripping for circuit remodeling. This review’s unique contribution is its integration of these classic extracellular pathways with the recently discovered intracellular complement system, or ‘complosome.’ We explore how the complosome offers a novel mechanistic framework linking complement to fundamental cellular processes, including metabolism and survival, particularly through its intricate connection with the master regenerative mTOR pathway. This highlights complement not as a simple inflammatory switch, but as a sophisticated signaling network. Understanding this duality is essential for developing therapies that selectively suppress complement-driven damage while enhancing its regenerative functions.

## The shifting paradigm of complement in axon regeneration

First characterized by Bordet and Gengou in 1901 for its antimicrobial functions, the complement system has long been regarded as a central component of innate immunity (1). Historically understood as a liver-derived, serum-based cascade activated via the classical, lectin, and alternative pathways (2, 3). These pathways converge to cleave the central component C3. This cleavage yields the anaphylatoxin C3a, which recruits immune cells, and the opsonin C3b, which “tags” targets for phagocytosis. C3b also propagates the cascade to cleave C5, generating the potent inflammatory mediator C5a and the C5b fragment, which initiates the lytic membrane attack complex (MAC). This duality—driving both inflammation (C3a/C5a) and clearance (C3b/MAC)—is fundamental to complement’s role in the nervous system. The scope of complement biology has since expanded dramatically. For instance, moving far beyond its role in immunity, the complement cascade, through proteins like C1q, C3, and C4, mediates essential synaptic pruning during normal brain development (4), and its dysregulation is linked to pathological synapse loss in neurodegenerative conditions like glaucoma and schizophrenia (5, 6). Recent research has revealed that key complement components, particularly C3 and C5, are active not only in extracellular immunity but also within cells (7, 8). This has led to the discovery of the ‘complosome,’ an intracellular complement system with critical roles in regulating fundamental cellular processes, including metabolism, survival, and oxidative stress responses (9–14). Importantly, the complosome also participates in sterile inflammation—an immune response triggered by damage-associated molecular patterns (DAMPs) (15) rather than pathogens. The interplay between the complosome and the NLRP3 inflammasome represents a critical nexus controlling cellular metabolism, inflammation, and cell survival.

The discovery of this intracellular system represents a significant paradigm shift. It reframes complement from a purely extracellular immune surveillance cascade to a fundamental intracellular regulator. This ‘complosome’ provides a direct mechanistic link between innate immunity and core cellular processes, such as metabolic status and survival decisions. For neuroimmunology, this concept is critical: it suggests complement can act *within* a neuron to directly influence its fate, rather than acting only as an external, environment-shaping force. This intracellular dimension is essential for understanding its dual role in sterile inflammation and repair.

A pressing challenge in neuroscience is the restoration of axonal integrity following trauma or in neurodegenerative disorders. While the peripheral nervous system (PNS) retains some regenerative capacity, neurons of the central nervous system (CNS) largely fail to regenerate their axons after injury. Axon disruption severs communication and triggers secondary degenerative processes in the affected neurons. Research has identified several intrinsic and extrinsic factors capable of promoting CNS axon regeneration. Key intrinsic strategies aim to reactivate developmental growth programs, primarily by downregulating the phosphatase and tensin homolog (Pten) (16–18). This is often combined with the upregulation of diverse pro-regenerative factors, such as specific microRNAs (19), cytoskeletal-associated proteins (20), transcription factors (21) and molecules involved in protein synthesis and mitochondrial function (22, 23). However, the clinical translation of these findings is often hindered; for example, Pten is a critical tumor suppressor, making its systemic inhibition a significant safety concern.

At the same time, extrinsic factors, such as fibronectin-derived peptides (24) and the potent inflammatory stimulus zymosan (25, 26), have also demonstrated pro-regenerative effects by modulating immune cell influx and cytokine release (24). However, the neurotoxicity associated with strong inflammatory stimuli like zymosan underscores a central paradox: inflammation can both facilitate and hinder neural repair. The complement system lies at the heart of this paradox. The complosome is directly linked to the very biological processes essential for neuroprotection and regeneration, including mitochondrial function, glucose metabolism, and antioxidant defense (12, 14, 27). While numerous complement components have been implicated in both promoting and inhibiting axon regeneration, the recent discoveries surrounding the intracellular complosome have added a critical new dimension to this duality. However, few reviews have comprehensively synthesized these classic extracellular and novel intracellular functions across both the peripheral and central nervous systems. Therefore, the central aim of this review is to bridge this gap. We will explore the multifaceted and often contradictory functions of complement components in the aftermath of neural injury, examining how this ancient defense system can both drive damaging inflammation and be harnessed to promote repair. These key dual functions are summarized in **Table 1**.

**Table 1:** The dual role of complement components in neuronal injury and repair.

Component	Role	Context/Model	Mechanism & key findings	Reference(s)
C5/C5a	Pro-Regenerative/Protective	PNS (Sciatic Nerve Crush)	Mediates early macrophage recruitment and debris clearance during Wallerian degeneration.	(28)
		CNS (Cortico-hippocampal neurons *in vitro*)	Directly increases the speed and length of axonal growth via the C5aR receptor.	(29)
		PNS (Human tooth pulp)	Stimulates fibroblasts to secrete NGF and BDNF, promoting neurite outgrowth.	(30, 31)
		CNS/PNS (General)	Drives recruitment and activation of phagocytic cells (microglia/macrophages) to clear inhibitory myelin and cellular debris.	(32–34)
	Detrimental/Inhibitory	CNS (Ischemia-Reperfusion)	Drives excessive neuronal autophagy and apoptosis by inhibiting the PI3K/Akt/mTOR pathway.	(35, 36)
		CNS (Huntington’s Disease Model)	Promotes neuroinflammation and neuronal death; C5aR1 antagonists are neuroprotective.	(37)
		PNS (Incisional Injury/Nociception)	Contributes to acute pain by directly sensitizing cutaneous nociceptors.	(38, 39)
C3/C3a/C3b	Pro-Regenerative/Protective	PNS (Sciatic Nerve Transection)	Mediates axotomy-induced synaptic stripping from motoneurons, improving functional recovery.	(40)
		CNS (Optic Nerve Crush)	Required for the recruitment of CR3-expressing microglia/monocytes to clear inhibitory myelin debris.	(41)
		CNS (Olfactory Disorder)	Necessary for neutrophil-dependent clearance and efficient replacement of olfactory receptor neurons.	(42)
	Detrimental/Inhibitory	CNS (Ocular Hypertension/Ischemia)	C3 and C3aR1 activation on microglia drives damaging neuroinflammation and retinal ganglion cell (RGC) death.	(43, 44)
		CNS (*in vitro*/Myelin Extracts)	Myelin-associated proteases cleave C3 into C3b, which directly inhibits neurite outgrowth and promotes neuron loss.	(45)
		CNS (Oxidative Stress)	Blocking the C3a/C3aR axis suppresses proinflammatory cytokines, microglial infiltration, and photoreceptor apoptosis.	(46)
NLRP3 Inflammasome	Pro-Regenerative/Protective	PNS (General)	Modulated NLRP3 signaling (via TSG-6) can promote the polarization of pro-regenerative M2 macrophages.	(47)
	Detrimental/Inhibitory	CNS/PNS (General)	Drives GSDMD-mediated pyroptosis, creating a pro-inflammatory environment hostile to repair.	(48, 49)
		CNS (Spinal Cord Injury)	A major driver of the secondary injury cascade; its inhibition reduces neuroinflammation and improves outcomes.	(50, 51)
		CNS (Optic Nerve Crush)	Genetic ablation of NLRP3 significantly delays RGC loss.	(52)
C1q/C4/MAC	Pro-Regenerative/Protective	CNS (Development)	Tags excess synapses for elimination by microglia, enabling healthy circuit refinement (synaptic pruning).	(4)
	Detrimental/Inhibitory	CNS (Alzheimer’s Disease)	C1q and C3 pathologically tag synapses for removal by microglia and astrocytes in response to Aβ and Tau.	(53, 54)
		CNS (Alzheimer’s Disease)	The MAC (terminal pathway) directly damages synapses, contributing to cognitive decline.	(55, 56)
		CNS (Schizophrenia)	Genetic variation leading to excessive C4A expression is linked to over-pruning of synapses and disease risk.	(5, 6)

## C5a-mediated inflammation and neurotrophic support in the PNS

The complement component C5 is cleaved by the C5 convertase enzyme complex, yielding the anaphylatoxin C5a and the C5b fragment, which initiates the formation of the membrane attack complex (MAC). This terminal pathway is itself a key modulator; recent work in a rat sciatic nerve injury model found that voluntary exercise prevented MAC formation at the injury site by upregulating the MAC inhibitor CD59, which correlated with neuroprotection and reduced pain (57). As a potent inflammatory mediator, C5a plays a dual role in the nervous system. On one hand, its activity can promote degeneration and tissue damage through excessive inflammation, contributing to the pathology of various neurological diseases. Conversely, controlled complement activation can be neuroprotective by facilitating the clearance of toxic protein aggregates and cellular debris, a beneficial effect in many neurodegenerative disorders (58).

The influence of complement extends to fundamental neural processes, including nociception. Local injection of C5a and C3a has been shown to produce mechanical and heat hyperalgesia by directly activating and sensitizing cutaneous nociceptors, implicating these fragments in the generation of acute pain (38). In a model of incisional injury, C5a levels were found to be significantly elevated in the affected skin, though not in the dorsal root ganglia (DRG) or spinal cord, suggesting a primarily peripheral role in this context (39). Recent work has provided a specific mechanism for complement-driven pain, showing that C5aR1 signaling *on Schwann cells* activates the NLRP1 inflammasome. This drives IL-1β release, subsequent macrophage recruitment, and TRPA1 activation, leading to widespread pain (59).

**Figure 1:**
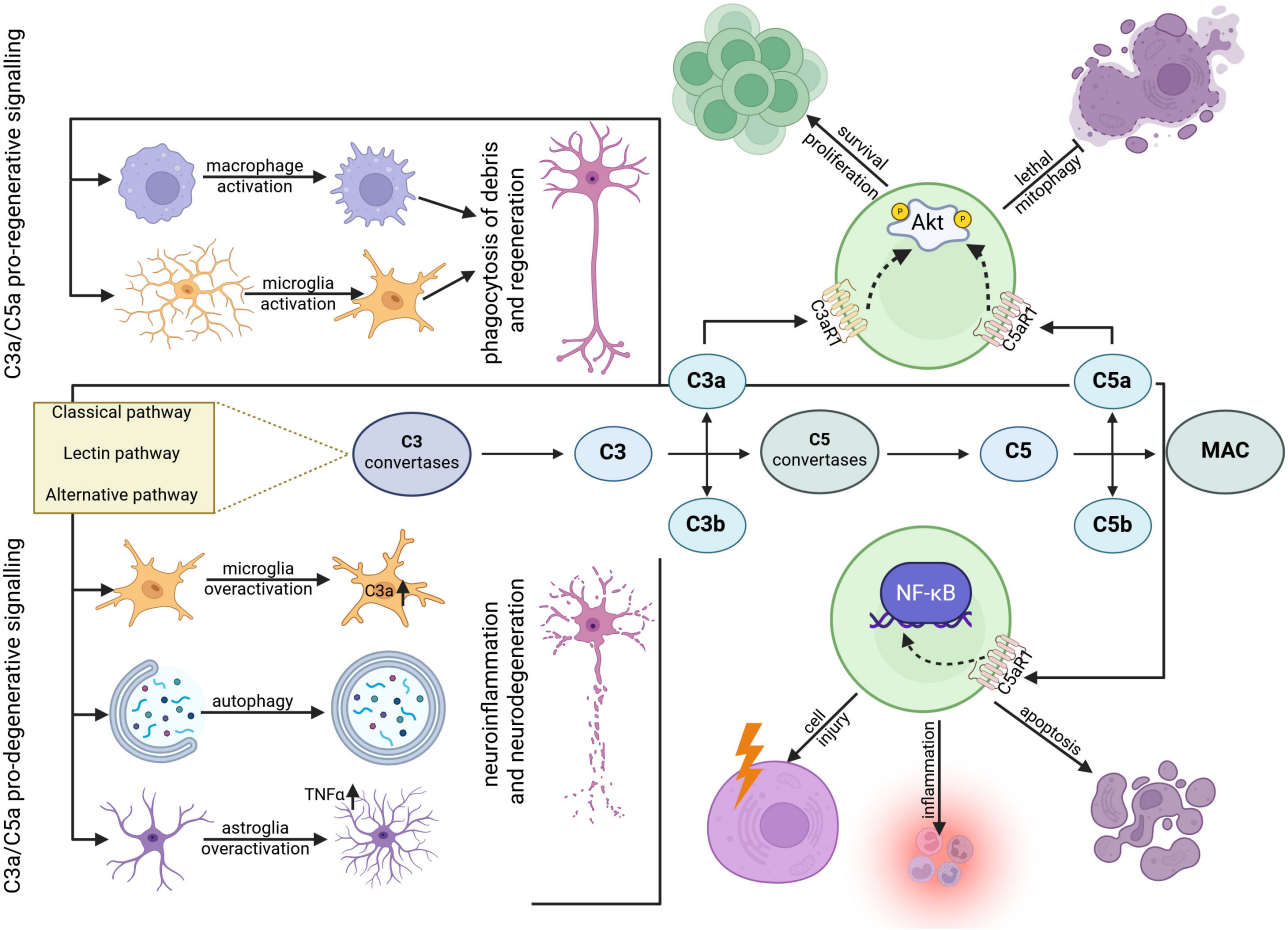
The dichotomous role of C3a and C5a signaling in neuronal fate after Injury. The anaphylatoxins C3a and C5a can elicit opposing outcomes depending on the cellular context and signaling intensity. (Upper panel) In a pro-regenerative context, C3a and C5a promote the recruitment and activation of microglia and macrophages. This enhances the phagocytic clearance of inhibitory myelin and cellular debris, creating an environment permissive for axon regeneration. Intracellularly, signaling through C3aR1 and C5aR1 can also drive Akt phosphorylation, promoting cell survival and proliferation while suppressing lethal mitophagy. (Bottom panel) Conversely, dysregulated signaling contributes to neurodegeneration. Excessive C3a and C5a can lead to the overactivation of microglia and astrocytes, resulting in the secretion of neurotoxic factors like TNF-α, which drives apoptotic pathways. C5aR1 signaling can also induce pathological autophagy and promote cell injury and inflammation via the NF-κB pathway.

One of the earliest studies investigating the role of C5 in the peripheral nervous system (PNS) utilized a sciatic nerve crush model in congenic mice lacking C5 (C5^(-)^). Compared to wild-type controls (C5^(+)^), the C5^(-)^ mice exhibited delayed macrophage recruitment and slower clearance of axonal and myelin sheath debris during the first 21 days post-injury. However, this delay did not affect the long-term recovery of motor function or the extent of axotomy-induced nerve cell death. These findings suggest that C5 and its derivatives are important for initiating the early inflammatory and clearance phases of Wallerian degeneration, likely by recruiting macrophages, but do not directly influence long-term functional restoration or central glial cell responses to peripheral injury (28) (**Figure 1**).

While these initial findings pointed to an indirect, immune-mediated role for C5, more recent research has revealed a direct effect of C5a on neurons. *In vitro* experiments on cultured cortico-hippocampal neurons with mechanically injured axons demonstrated that treatment with C5a (50–100 nM) significantly increased the speed and length of axonal growth. This pro-regenerative effect was dependent on the C5a receptor (C5aR), as co-treatment with a C5aR antagonist (PMX53) blocked C5aR phosphorylation and inhibited axon growth. The study also confirmed the co-localization of a neural marker with C5aR in both the primary neuronal cultures and in the gray matter of the spinal cord, supporting a direct C5a-neuron interaction (29).

Further evidence for a positive role of C5a in PNS regeneration comes from studies using human tooth pulp. One investigation demonstrated that human pulp fibroblasts express C5aR and that its expression increases in response to carious injury *in vivo* and lipoteichoic acid stimulation *in vitro*. This was followed by local C5a activation, which in turn stimulated the secretion of Nerve Growth Factor (NGF) from the fibroblasts, promoting prominent neurite outgrowth (31). A subsequent study by the same group confirmed that the C5a-C5aR interaction also activates the secretion of Brain-Derived Neurotrophic Factor (BDNF), guiding neuronal growth towards the site of injury (30). Together, these studies highlight a key mechanism where C5a promotes axon outgrowth in the PNS by inducing the local release of essential neurotrophic factors.

In summary, the role of C5a in the PNS exemplifies the central theme of this review: its function is highly context-dependent. The evidence clearly distinguishes between at least three mechanisms: (1) an indirect, immune-mediated pathway essential for early Wallerian degeneration, where C5a recruits macrophages to clear debris (28); (2) a direct, pro-regenerative pathway on neurons, where C5aR signaling at specific concentrations (e.g., 50–100 nM) promotes axonal growth (29); and (3) an indirect, neurotrophic pathway, where C5a stimulates local fibroblasts to secrete NGF and BDNF (30). The outcome—detrimental inflammation and pain (38) versus controlled, pro-regenerative activity—appears to depend on the precise location (e.g., peripheral nociceptors vs. the nerve trunk), concentration, and timing of C5a signaling. This complexity underscores that broad C5a/C5aR1 inhibition, while beneficial in some chronic inflammatory contexts, may inadvertently block essential repair mechanisms. Future therapies may need to modulate, rather than ablate, this pathway to uncouple its beneficial debris-clearance and neurotrophic functions from its detrimental inflammatory effects.

## C3/C3a as a context-dependent mediator of neurodegeneration and repair

Within the complement cascade, the central component C3 is cleaved by C3 convertases into the opsonin C3b and the anaphylatoxin C3a. In the canonical view, C3a acts as an agonist for its receptor, C3aR, stimulating chemotaxis and immune cell activation. Like C5a, C3a has specific roles within the nervous system that extend beyond its classic immune functions. Both C3a and C5a are strongly and consistently associated with pain states (60). Furthermore, dysregulation of C3a signaling contributes to the pathogenesis of several neurological diseases, including stroke (61) and multiple sclerosis (62), while also playing a role in fundamental processes such as adult neurogenesis (63, 64). This section will focus specifically on the contribution of C3 and C3a to the mechanisms of axon regeneration.

## C3/C3a in the PNS: a pro-regenerative role

In the peripheral nervous system (PNS), the role of C3 appears to be beneficial for recovery. Experiments using C3-deficient (C3^(-)^) mice following sciatic nerve transection demonstrated that C3, but not C1q, is a key mediator of axotomy-induced synaptic stripping from injured motoneurons. The C3^(-)^ mice showed a reduced loss of synaptic terminals and an increased expression of GAP-43 mRNA, a marker for axonal growth, which correlated with improved functional recovery. This identifies C3 as a critical element for synaptic remodeling, an essential step in the regenerative process following peripheral nerve injury (40). Further evidence for a direct, beneficial role comes from recent work identifying distinct localizations for C3aR (on glial paranodes of large-myelinated fibers) and C5aR1 (on small unmyelinated fibers) in human and mouse peripheral nerves. Importantly, C3aR activation was shown to enhance neuronal excitability in large fibers, suggesting a novel mechanism for complement in modulating PNS neuronal function beyond just synaptic remodeling (65). This suggests that in the PNS, C3-mediated synaptic remodeling is a critical and beneficial component of a successful, spatially-controlled repair program.

## C3/C3a in the CNS: a dichotomous function

In the central nervous system (CNS), however, the role of C3 is more complex and appears highly context dependent. Several studies indicate that C3 activation can be detrimental. In models of ocular hypertension, where complement components are locally synthesized (66–68), the absence of C3 was shown to delay axonal degeneration and retinal ganglion cell (RGC) death following ischemia-reperfusion injury (43). Similarly, another study using a murine ocular hypertension model identified C3aR1 as a key driver of damaging neuroinflammation. In this model, high levels of C3aR1 were expressed on microglia and infiltrating myeloid cells, positioning them as the primary mediators of its effects and establishing C3aR1 as a major regulator of microglial reactivity, likely through interactions with IL-10 signaling and other immune pathways (44). Similarly, in a model of retinal degeneration induced by oxidative stress, blocking the C3a/C3aR axis suppressed proinflammatory cytokine release, microglial infiltration, and photoreceptor apoptosis. These findings suggest that under conditions of ischemic or oxidative stress, C3 and C3a promote retinal inflammation and degeneration (46). Further evidence for a direct inhibitory role comes from a study showing that protein C3 negatively regulates axonal growth *in vivo* and *in vitro*. The addition of C3 to cultured neurons exposed to inhibitory myelin extracts tripled both neurite outgrowth inhibition and neuron loss. Mechanistically, the study revealed that myelin-associated serine proteases cleave C3, and experiments with purified fragments suggested that C3b, rather than C3a, is the fragment responsible for this growth-inhibitory and neurotoxic activity (45).

In contrast to its inhibitory effects, other studies suggest that C3 plays an essential and beneficial role in facilitating CNS repair. Following optic nerve crush (ONC), an injury model of mechanical axotomy, C3 and C1q were required for the recruitment and activation of microglia/monocytes expressing the complement receptor CR3. These cells were essential for clearing inhibitory myelin debris, a critical step for facilitating RGC axon regeneration (41). Further supporting a local regulatory role, another ONC study found that the complement inhibitor Clusterin was upregulated in astrocytes at the lesion site, suggesting an endogenous defense mechanism to control complement activation. This study also implicated reactive microglia as a key local source of C3 (69).

The pro-regenerative function of C3 extends to other parts of the CNS. In a model of olfactory disorder, C3-deficient mice showed delayed recovery and maturation of olfactory receptor neurons (ORNs). This was linked to impaired, neutrophil-dependent clearance of undesired ORNs, indicating C3 is necessary for efficient neuronal replacement and regeneration in this system (42). Furthermore, in a spinal cord injury (SCI) model, C3 deficiency was found to suppress astrocyte activation and TNF-α expression, thereby reducing neuroinflammation and improving axonal regeneration (70).

This C3-dependent process of synaptic remodeling, while beneficial after acute injury, is now understood to be pathologically hijacked in neurodegenerative conditions like Alzheimer’s disease (AD). Seminal studies have demonstrated that C1q and C3 “tag” synapses for elimination by CR3-expressing microglia in response to soluble β-amyloid (Aβ) oligomers and pathological Tau. This process drives early synapse loss, a key correlate of cognitive decline in AD (53). This C3a/C3aR signaling is also implicated in the transition of microglia from a homeostatic (e.g., P2ry12+) to a detrimental disease-associated microglia (DAM) phenotype, which exacerbates synapse elimination.

Further research has revealed that this process is amplified by a critical astrocyte-microglia crosstalk: reactive microglia release cytokines (e.g., IL-1α, TNF, C1q) that induce astrocytes to produce C3. This astrocyte-derived C3 then “tags” synapses for removal by microglial CR3, creating a feed-forward loop of pathology, and inhibition of C1q or C3 can rescue this deficit (54, 71). Beyond the classical pathway, the terminal pathway has also been implicated, with the MAC directly damaging synapses. Consequently, blocking MAC formation, either by targeting C7 with an antibody or through genetic deletion of C6, has been shown to reduce synapse loss and improve cognition in AD mouse models (55, 56). These findings highlight a critical contrast: while complement-mediated phagocytosis can be beneficial for clearing debris like amyloid plaques, its dysregulation in AD leads to the pathological destruction of essential neural circuits.

In summary, the role of C3 is heavily dependent on the nervous system branch and the specific injury context. In the PNS, its function in synaptic stripping is clearly pro-regenerative. In the CNS, this function is dichotomous (**Figure 1**). The “switch” from protective to destructive appears to be dictated by the trigger: in mechanical injury (e.g., ONC), C3 is indispensable for beneficial debris clearance, whereas in pathological conditions (e.g., ischemia, oxidative stress, or AD), C3 signaling drives neuroinflammation and synapse loss. This duality between beneficial phagocytic tagging (debris) and pathological tagging (synapses) suggests that the *location* and *context* of C3 activation are paramount. While the evidence reviewed here is primarily extracellular, these divergent outcomes may also be influenced by the intracellular complosome. It is plausible that the balance between extracellular C3 activity (driving inflammation/phagocytosis) and intracellular C3 signaling (promoting metabolic homeostasis, as discussed later) is a key determinant of the ultimate cellular response.

## The intersection of complement, apoptosis, and autophagy in neuronal fate regulation

What determines a neuron’s fate after axonal disruption—be it from acute trauma or chronic disease—remains a key focus in neuroscience research. The severance of an axon from its target disrupts the neural circuit, depriving the neuron of essential stimuli and trophic support, which often triggers apoptotic or necrotic cell death pathways. Concurrently, cellular maintenance processes like autophagy, an intracellular degradation system, become critically important. Autophagy can support cell growth and survival by recycling components and removing damaged organelles (72, 73). A specialized form, mitophagy, targets dysfunctional mitochondria, providing a key defense against oxidative stress and aging (74). However, this process is a double-edged sword; while initially protective, excessive or “lethal mitophagy” can drive the cell toward programmed death (75). Numerous studies have implicated complement receptors in the regulation of these fundamental survival and death pathways, with a particular emphasis on neurons.

On a molecular level, the ligation of the G-protein-coupled receptors C3aR1 and C5aR1 can activate pro-survival signaling. In immune cells, for example, receptor ligation can activate the PI-3Kγ/Akt (PKB) pathway, which promotes proliferation and survival (76–78). This signaling can also be protective by suppressing lethal mitophagy (79). Conversely, the outcome is context-dependent: in other settings, C5a-C5aR signaling can also induce autophagy-mediated apoptosis (80). While these studies establish a clear link between complement receptors and cell fate decisions in immune cells, parallel mechanisms are now being uncovered in the nervous system.

The eye, an extension of the CNS, is considered an immune-privileged site where the complement system is normally maintained at a low, tolerogenic level (81, 82), although all retinal cells are capable of expressing complement components (83). However, under pathological conditions, this balance can be disrupted. In a glaucoma model, the death of retinal ganglion cells (RGCs) was associated with the activation of the classical complement pathway and a corresponding inflammatory cytokine response (84). In another study using a light-induced retinal damage model, microglia were identified as a primary source of C3. The deposition of C3 in the outer nuclear layer activated the complement cascade, exacerbating photoreceptor cell death (85).

The C5a receptor, C5aR1, has emerged as a particularly critical player in neuronal death. In brain ischemia-reperfusion (I/R) injury, for example, autophagy switches from protective to detrimental (86). Complement C5a generated by ischemic neurons appears to drive this pathological switch via C5aR1 (87–89). The mechanism involves modulating key survival cascades. In a cardiac arrest model, C5a-C5aR1 engagement drove excessive neuronal autophagy by inhibiting the pro-survival PI3K/Akt/mTOR pathway (35). In a separate cerebral ischemia model, C5aR1 inhibition was neuroprotective by blocking the pro-inflammatory NF-κB signaling pathway, which in turn reduced cell injury and apoptosis (36).

Similarly, in a neurotoxin-induced model of Huntington’s disease, the administration of C5aR1 antagonists significantly reduced striatal lesion size, apoptosis, and neuroinflammation. While the antagonists had no direct effect on neuronal cultures, this finding pointed towards an indirect, inflammation-driven mechanism of neuroprotection *in vivo*, where blocking C5aR1 on immune cells prevents the cascade of events leading to neuronal death (37).

In summary, C5aR1, is a pivotal regulator of neuronal fate after CNS injury. Its activation on both neurons and immune cells can trigger pathological autophagy and apoptosis. The mechanism hinges on the modulation of key intracellular signaling cascades, including the PI3K/Akt/mTOR and NF-κB pathways. This positions complement receptors as critical therapeutic targets for mitigating neurodegeneration following CNS injury.

## C5a-C5aR1 axis in immune cell recruitment and debris clearance

A critical determinant of successful axon regeneration is the effective clearance of inhibitory debris from the injury site. Following trauma to the nervous system, the local microenvironment becomes hostile to regrowth due to the accumulation of myelin debris and the formation of a glial scar, which is rich in inhibitory chondroitin sulfate proteoglycans (CSPGs) like aggrecan and brevican (90–92). These molecules create a potent physical and chemical barrier that blocks regeneration, even if neurons have initiated a growth program. Therefore, the recruitment and activation of phagocytic cells, primarily monocytes and macrophages from the circulation, are essential for clearing this inhibitory environment and creating a permissive path for regrowing axons (93).

The complement system is a key driver of this necessary inflammatory response. The anaphylatoxins C3a and C5a are powerful chemoattractants that establish a chemical gradient, guiding immune cells to the site of injury through their respective receptors, C3aR and C5aR (94). It is important to note that C5a does not act in isolation; it works in concert with other chemotactic systems, such as the MCP-1/CCR2 and CX3CL1/CX3CR1 axes, to orchestrate the full immune response. In the context of the nervous system, C5a has been shown to protect neurons by promoting microglial phagocytosis of cellular debris (32, 33, 95). This function is underpinned by specific intracellular mechanisms, as mitochondrial C5aR1 signaling is required for the pro-inflammatory and phagocytic activity of macrophages (96). However, this response must be tightly controlled; while essential for initial debris clearance, persistent complement-mediated inflammation can become chronic and detrimental, contributing to secondary injury.

The importance of this axis in nerve repair is highlighted in a facial nerve crush model. Transcriptomic analysis revealed that C5ar1 was among 39 genes significantly upregulated in the injured nerve trunk, contributing to enhanced leukocyte adhesion and phagocytosis, which promoted repair. The expression of C5ar1 was rapidly upregulated at 6 hours post-injury, peaked at 4 days, and returned to baseline by 7 days. This temporal expression pattern mirrors that previously reported following spinal cord injury, suggesting that the C5a-C5aR1 axis plays a conserved and critical role in orchestrating the early immune response to trauma in both the peripheral and central nervous systems (34, 97). This concept of a dynamically shifting cellular landscape has been confirmed by recent single-cell transcriptomics, which mapped the precise temporal infiltration of immune cells (peaking early) followed by the proliferation of repair Schwann cells. Notably, more severe injuries showed delayed glial recovery, reinforcing the importance of this early immune-glial coordination (98). This transient expression profile is critical: the C5a-C5aR1 axis is beneficial when it is an acute, self-limiting response. The “switch” from beneficial phagocytosis to a harmful, chronic inflammatory state likely occurs when this signaling fails to resolve, leading to sustained immune cell activation and a neurotoxic environment.

This section highlights the critical importance of *temporal control* for the C5a-C5aR1 axis. Its beneficial, pro-regenerative role in debris clearance is an acute, transient event. This presents a significant translational challenge: therapeutic strategies must be designed to either promote this early, beneficial response or, more likely, to inhibit the pathway *after* the initial clearance phase is complete. Temporally-controlled inhibition could prevent the “switch” to chronic, detrimental inflammation, thereby preserving the regenerative benefits while mitigating the secondary, complement-driven damage.

## The NLRP3 inflammasome as a central regulator of post-injury inflammation and regeneration

The NOD-, LRR-, and pyrin domain-containing protein 3 (NLRP3) inflammasome is a multiprotein complex that serves as a critical sensor for cellular danger and a key driver of sterile inflammation. Its close interaction with the intracellular complement system, or complosome, places it at a crucial nexus between innate immunity and cellular metabolism. Given its central role, the function of the NLRP3 inflammasome has been extensively investigated in the context of nervous system injury, where evidence reveals it plays a complex and often contradictory role in regulating regenerative outcomes.

A significant body of evidence points to a detrimental role for NLRP3 activation following neural trauma. Foundational work demonstrated that the complement membrane attack complex (MAC) can directly disrupt the myelin sheath and trigger a macrophage-led attack, leading to demyelination (99). More recent studies have linked this to NLRP3, which, upon activation, recruits caspase-1 to cleave gasdermin D (GSDMD). The resulting N-terminal fragment of GSDMD forms pores in the cell membrane, leading to a lytic, pro-inflammatory cell death known as pyroptosis (100, 101). In a sciatic nerve transection model, GSDMD-driven pyroptosis was shown to be dependent on NLRP3 activity and responsible for creating a pro-inflammatory macrophage environment that is hostile to repair (48). This mechanism is conserved across the nervous system, as NLRP3 activation also drives delayed pyroptosis of retinal cells following ONC (49).

In the context of SCI, the NLRP3 inflammasome is recognized as a major driver of the deleterious secondary injury cascade (50). Consequently, strategies aimed at inhibiting its activity have shown significant therapeutic promise. For example, duraplasty with custom-fabricated biomaterial membranes was found to promote axon growth by decreasing NLRP3 expression and reducing the infiltration of pro-inflammatory macrophages at the lesion site (102). Similarly, pharmacological inhibition of the NLRP3 inflammasome with the selective inhibitor OLT1177 conferred significant neuroprotection and improved functional outcomes after SCI (51). The detrimental role of NLRP3 is further underscored in the visual system, where genetic ablation of NLRP3 significantly delayed RGC loss after optic nerve crush (52). This effect can also be achieved indirectly, as interventions that reduce NLRP3 activation and subsequent IL-1β/IL-18 production—such as deleting arginase 2 or inhibiting HMGB1—have been shown to enhance neuroprotection and axonal sprouting (103, 104). The upstream regulation of this pathway is also critical, as molecules like Complement Factor H-related protein 3 (FHR-3) can activate the NLRP3 inflammasome, while others, like the E3 ubiquitin ligase Synoviolin, can suppress it (9, 105).

A key aspect of this pathway, however, is how it is activated. Complement components themselves are emerging as critical upstream drivers of NLRP3. The C5a-C5aR1 axis, for instance, can provide the priming signal (Signal 1) for NLRP3 activation by inducing mitochondrial ROS. Furthermore, signaling through C5aR1 can also provide the activation signal (Signal 2) by promoting potassium efflux, a key trigger for inflammasome assembly. The intracellular complosome is also implicated, as cell-intrinsic C3 activation is required for the metabolic reprogramming that fuels inflammasome-driven inflammation (11). This positions complement not just as a parallel inflammatory pathway, but as a direct, DAMP-like signal that can initiate and amplify the entire NLRP3-pyroptosis cascade.

**Figure 2:**
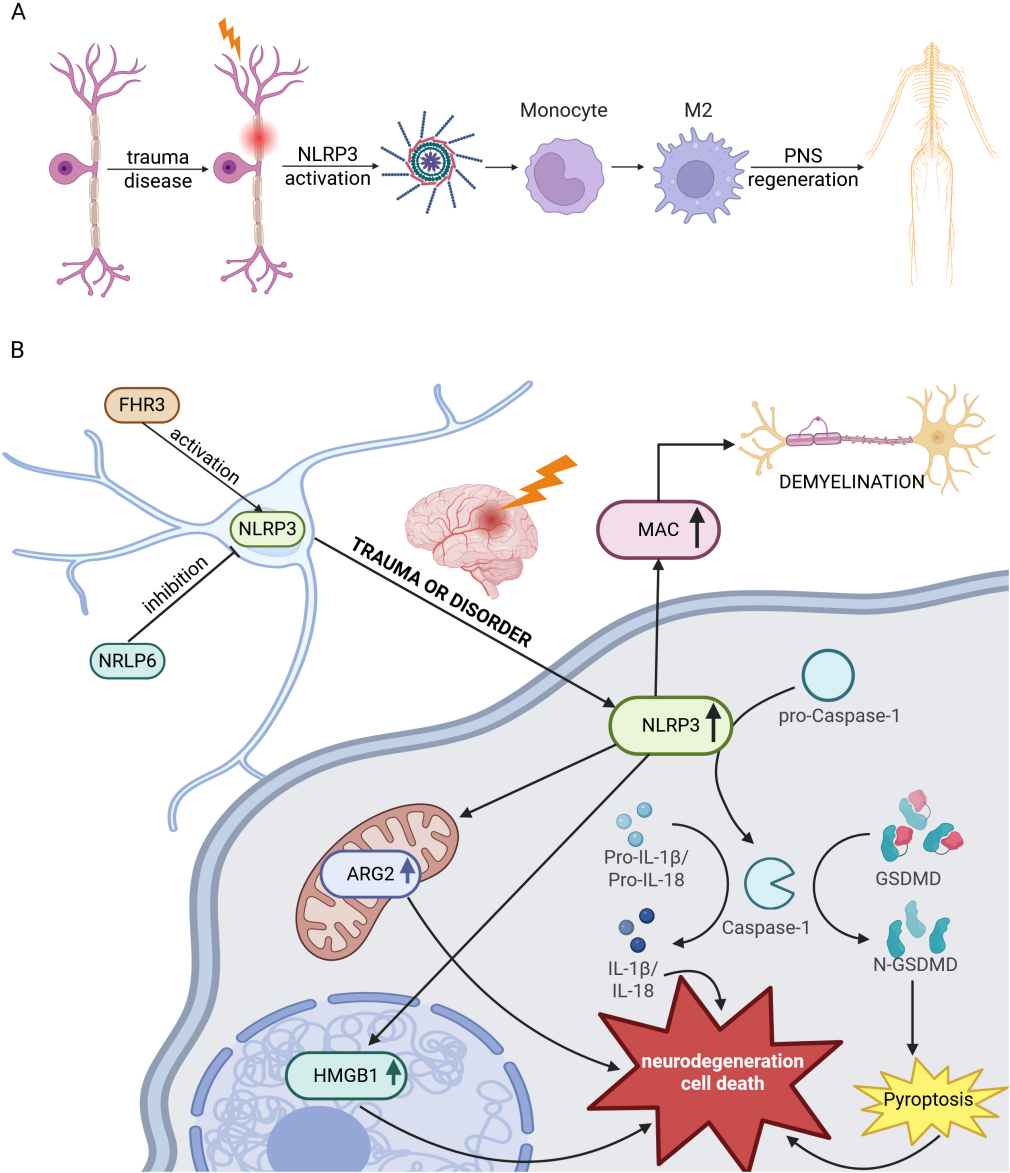
The context-dependent role of the NLRP3 inflammasome in the peripheral and central nervous systems. The functional outcome of NLRP3 inflammasome activation differs significantly between the PNS and CNS following injury. **(A)** In the PNS, NLRP3 signaling has been shown to support regeneration by promoting the differentiation of macrophages toward a pro-regenerative M2 phenotype. **(B)** In the CNS, NLRP3 activation is largely associated with neurodegeneration. Upstream regulators like FHR-3 can activate the inflammasome, while proteins such as NLRP6 may inhibit its activity. Following traumatic injury, NLRP3 activation drives multiple detrimental pathways, including caspase-1-dependent pyroptosis via GSDMD cleavage. Recent evidence indicates this activation can be triggered by the deposition of the membrane attack complex (MAC), which contributes to demyelination. Furthermore, it triggers the production of IL-1β and IL-18 and the activation of ARG2 and HMGB1, all of which contribute to a neurotoxic environment, glial activation, and neuronal cell death.

Despite the overwhelming evidence of its destructive capacity, some studies suggest that NLRP3 activity can be beneficial in specific contexts. The polarization of macrophages towards a pro-regenerative M2 phenotype is essential for PNS repair (106) (**Figure 2A**). In one study, exosomes derived from LPS-preconditioned mesenchymal stem cells promoted this M2 switch by transferring the protein TSG-6, which modulated the NF-κB/NLRP3 signaling pathway in macrophages (47). This suggests that, rather than being purely detrimental, NLRP3 signaling may be necessary for instructing specific immune cell fates. Further complicating the picture, other related proteins like NLRP6 have been shown to contribute to peripheral nerve recovery independently of the canonical inflammasome, potentially by attenuating its activity (107).

In conclusion, the role of the NLRP3 inflammasome in neural repair is decidedly dualistic, as conceptualized in **Figure 2**. This duality raises critical questions for future research. Most evidence indicates that over-activation drives a destructive cycle of pyroptosis (**Figure 2B**), yet context-specific signaling may be required for beneficial M2 macrophage polarization (**Figure 2A**). A key translational challenge is therefore to uncouple these two outcomes. Future studies should aim to identify the specific upstream signals—be it complement, DAMPs, or other factors—that determine whether NLRP3 activation triggers beneficial M2 polarization or detrimental pyroptosis. Understanding this “switch” will be essential for designing nuanced therapies that can selectively ablate the inflammasome’s destructive capacity while preserving its potential role in orchestrating repair.

## The intracellular complosome and its link to the mTOR pathway

The mechanistic target of rapamycin (mTOR) pathway is a master regulator of cell growth, metabolism, and survival. Its role in the nervous system is particularly critical, as inhibition of its negative regulator, the tumor suppressor Pten, is one of the most potent strategies known for promoting axon regeneration (18, 23, 108). Emerging evidence now reveals a significant and complex interplay between this central regenerative pathway and the complement system, particularly the intracellular complosome.

Pioneering work in immunology has established a direct link between intracellular complement activation and mTOR. The continuous, cell-intrinsic generation of C3 fragments within T cells is required to sustain tonic mTOR signaling, which is essential for their homeostatic survival (109). Upon T-cell activation, this intracellular C3 cleavage is further enhanced, driving the metabolic shift toward glycolysis and oxidative phosphorylation necessary for effector differentiation. This is achieved by boosting nutrient influx and activating the mTOR complex 1 (mTORC1), demonstrating that the complosome acts as a fundamental hub for cellular metabolic reprogramming (110–112).

The anaphylatoxin C5a also directly modulates the mTOR signaling cascade, though its effects appear highly context dependent. For instance, in lupus effector T cells, C5a enhances Akt activity—a key kinase downstream of mTOR—thereby promoting cell migration (113). Similarly, C5aR1 signaling is required to activate mTOR and drive the differentiation of T follicular helper cells (114). Conversely, other studies report an opposing relationship. In intracranial aneurysms, mTOR inhibition leads to an *upregulation* of C5aR1, which in turn recruits neutrophils and drives inflammation (115). Furthermore, as discussed previously, C5a signaling through C5aR1 can induce pathological neuronal autophagy by *inhibiting* the pro-survival PI3K/Akt/mTOR pathway during ischemia-reperfusion injury (35). The mTOR inhibitor rapamycin has itself been shown to be neuroprotective by suppressing glial activation and reducing the expression of inflammatory factors like NLRP3, highlighting the therapeutic potential of modulating this nexus of inflammation and metabolism (116).

Beyond these direct interactions, complement components can also influence mTOR signaling indirectly through complex crosstalk with other receptor systems. A key example involves the vascular endothelial growth factor receptor 2 (VEGFR2), a receptor tyrosine kinase. For VEGFR2 to become fully activated and signal effectively, it requires simultaneous input from C3aR1, C5aR1, and the IL-6 receptor complex. In this signaling hub, the inhibition of any one component suppresses the others, preventing the downstream activation of key pathways involving Akt and STAT3. This indicates that complement receptors do not act in isolation but are part of larger “supercomplexes” that integrate diverse signals to control fundamental cellular decisions (117).

**Figure 3:**
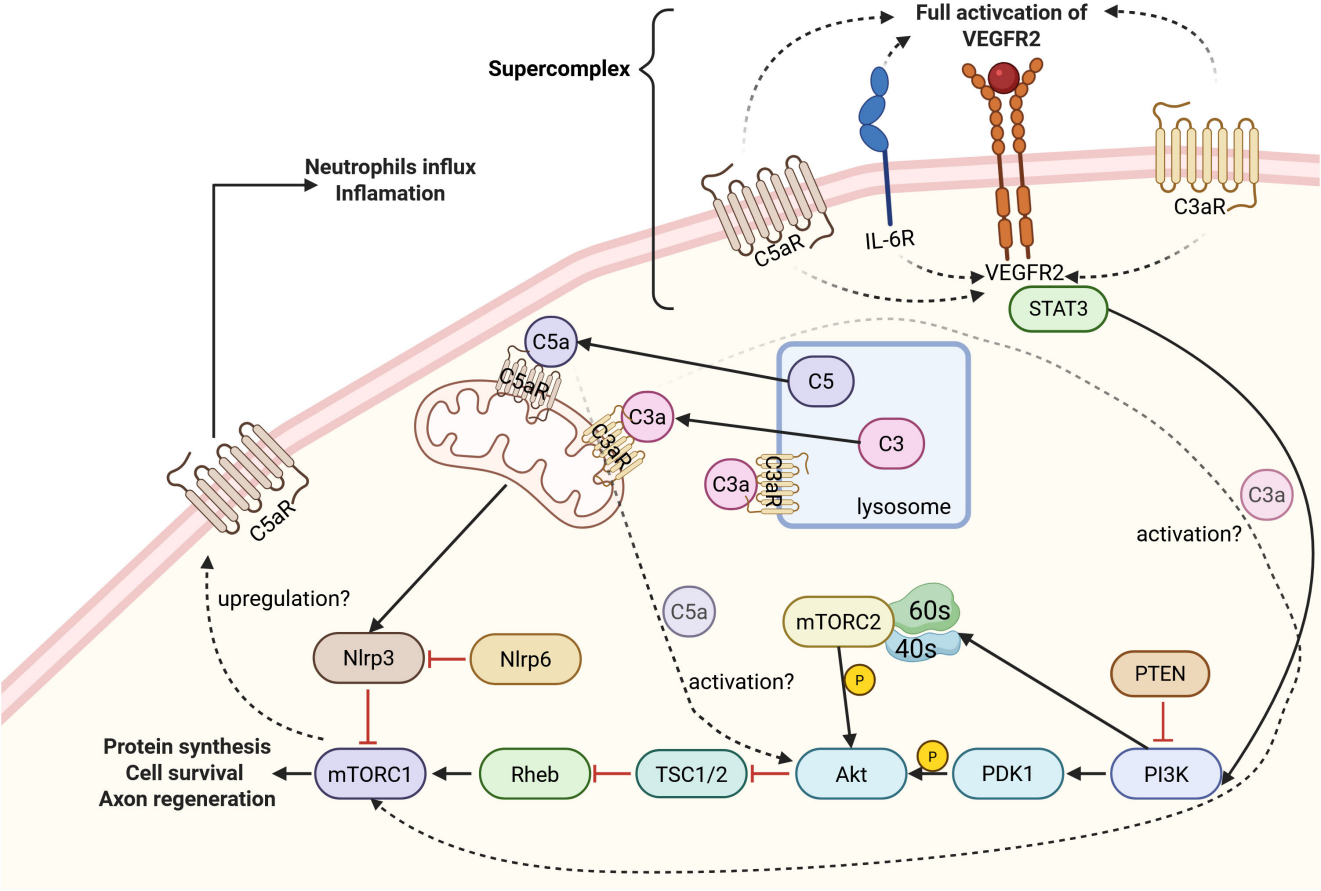
A proposed model for the regulation of the PI3K/Akt/mTOR pathway by complement signaling. Evidence suggests the formation of a putative signaling supercomplex involving C3aR1, C5aR1, IL-6R, and VEGFR2. Within this proposed complex, simultaneous input from the complement and IL-6 receptors appears necessary for the full activation of VEGFR2, leading to downstream STAT3 phosphorylation and engagement of the pro-survival PI3K/Akt/mTOR pathway. In addition to this receptor crosstalk, intracellular complement components may also directly influence this cascade. Intracellular C3a has been shown to activate mTORC1, while intracellular C5a may promote Akt activation. A potential positive feedback loop has also been proposed, wherein mTORC1 activation leads to the upregulation of C5aR1, which could enhance inflammatory responses such as neutrophil recruitment. Dashed lines and question marks in the schematic indicate pathways that are hypothesized or require further investigation.

In summary, the complement system is deeply intertwined with the mTOR pathway, as conceptualized in our proposed model (**Figure 3**). However, the evidence is bifurcated, and it is critical to be transparent about what is known versus what is hypothesized. The most robust evidence for complosome-mTOR crosstalk comes from (a) immune cells, where intracellular C3 is essential for sustaining pro-survival mTORC1 signaling and metabolic reprogramming. In contrast, (b) the direct evidence in neural cells is more limited but highly suggestive of a different, context-dependent role. The key finding in neurons shows extracellular C5a-C5aR1 inhibiting the PI3K/Akt/mTOR pathway to drive pathological autophagy (35). This creates a critical knowledge gap: how does the intracellular complosome, which is pro-mTOR in T-cells, behave within an injured neuron? It is highly plausible that this internal complosome-mTOR axis could be harnessed to support the high metabolic demands of axon regeneration. Dissecting the divergent roles of this intracellular (potentially pro-regenerative) versus the extracellular (often pathological) complement-mTOR axis is a crucial future direction for the field.

## A perspective on complement in neural repair

The evidence reviewed here demonstrates that the complement system is a critical, multifaceted regulator of neural fate after injury. The central challenge this review addresses is the ‘complement paradox’: under what biological contexts is its activation beneficial versus harmful? The literature suggests a clear answer: beneficial, pro-regenerative functions (e.g., debris clearance, synaptic stripping) are typically acute, transient, and spatially controlled. In contrast, detrimental, neurodegenerative functions (e.g., pyroptosis, neuroinflammation, pathological synapse loss) are driven by chronic, unresolved, or excessive activation, often in response to pathological triggers like ischemia or amyloid.

The discovery of the intracellular complosome provides a novel mechanistic framework for this duality, connecting it directly to fundamental cell-intrinsic processes like metabolism (via mTOR) and autophagy. Ultimately, the complement system emerges not as a simple “on/off” inflammatory switch, but as a sophisticated signaling network. This complexity demands that future therapeutic strategies move beyond broad inhibition. A more nuanced, forward-looking approach is required, which could include: (1) selective C5aR1 antagonists with specific temporal dosing to block chronic neuroinflammation while sparing acute, beneficial debris clearance; (2) targeted NLRP3 inhibitors to specifically prevent pyroptosis without globally suppressing inflammation; or (3) novel metabolic modulators designed to enhance the pro-regenerative intracellular complosome–mTOR signaling axis. Dissecting these context-dependent pathways, along with identifying reliable biomarkers to predict outcomes, is the key to unlocking complement’s full therapeutic potential.
